# Differences in Ultrasonic Vocalizations between Wild and Laboratory California Mice (*Peromyscus californicus*)

**DOI:** 10.1371/journal.pone.0009705

**Published:** 2010-04-01

**Authors:** Matina C. Kalcounis-Rueppell, Radmila Petric, Jessica R. Briggs, Catherine Carney, Matthew M. Marshall, John T. Willse, Olav Rueppell, David O. Ribble, Janet P. Crossland

**Affiliations:** 1 Department of Biology, University of North Carolina at Greensboro, Greensboro, North Carolina, United States of America; 2 Department of Education Research and Methodology, University of North Carolina at Greensboro, Greensboro, North Carolina, United States of America; 3 Department of Biology, Trinity University, San Antonio, Texas, United States of America; 4 Peromyscus Genetic Stock Center, University of South Carolina, Columbia, South Carolina, United States of America; University of Sussex, United Kingdom

## Abstract

**Background:**

Ultrasonic vocalizations (USVs) emitted by muroid rodents, including laboratory mice and rats, are used as phenotypic markers in behavioral assays and biomedical research. Interpretation of these USVs depends on understanding the significance of USV production by rodents in the wild. However, there has never been a study of muroid rodent ultrasound function in the wild and comparisons of USVs produced by wild and laboratory rodents are lacking to date. Here, we report the first comparison of wild and captive rodent USVs recorded from the same species, *Peromyscus californicus*.

**Methodology and Principal Findings:**

We used standard ultrasound recording techniques to measure USVs from California mice in the laboratory (*Peromyscus* Genetic Stock Center, SC, USA) and the wild (Hastings Natural History Reserve, CA, USA). To determine which California mouse in the wild was vocalizing, we used a remote sensing method that used a 12-microphone acoustic localization array coupled with automated radio telemetry of all resident *Peromyscus californicus* in the area of the acoustic localization array. California mice in the laboratory and the wild produced the same types of USV motifs. However, wild California mice produced USVs that were 2–8 kHz higher in median frequency and significantly more variable in frequency than laboratory California mice.

**Significance:**

The similarity in overall form of USVs from wild and laboratory California mice demonstrates that production of USVs by captive *Peromyscus* is not an artifact of captivity. Our study validates the widespread use of USVs in laboratory rodents as behavioral indicators but highlights that particular characteristics of laboratory USVs may not reflect natural conditions.

## Introduction

There has been extensive laboratory research on rodent USVs within the superfamily Muroidea [1], especially in laboratory mice (*Mus* spp) and rats (*Rattus* spp), which serve as mammalian non-human models in most areas of biological research. *Mus* and *Rattus* predictably produce USVs in the laboratory and their USV patterns are used as a phenotypic marker in behavioral assays [2]. All muroid rodents examined have been shown to produce USVs as juveniles and/or adults [1]. In the laboratory, muroid rodent USVs are suggested to have a communication function which is supported by observations that they are structured signals that cause predictable behavioral responses in recipients [3]. In *Rattus*, USVs are associated with positive and negative affective states [4]. In adult *Mus*, USVs are associated with male-female [5] and female-female social interactions [4].

Despite valuable research on USVs in laboratory rodents, it is unclear how USVs function in the wild. Although functions have been attributed to USVs produced in the laboratory, it is important to understand them in the wild because only then can the social context of USV evolution be understood. This problem was highlighted 30 years ago when W.J. Smith stressed that understanding the evolutionary significance of USVs in laboratory rodents was a “serious matter” given artificial social contexts in laboratories [6]. Nevertheless, there has never been a study of muroid rodent ultrasound function in the wild nor a comparison between USVs produced by wild and laboratory rodents.

The genus *Peromyscus* (deer mice; Muroidea, Cricetidae) contains over fifty species, has a wide geographic distribution over most of North and Central America, is common in almost every terrestrial habitat within its range, and displays a substantive range of genetic, morphological, behavioral, and physiological variation [7], making *Peromyscus* a widely used model for evolution, conservation, genetics, and behavior research. In particular, the monogamous California mouse (*P. californicus*) is a laboratory model for parental behavior [8], [9], [10], [11], [12] and pair bonding [13], [14], [15], [16], [17].

**Figure 1:**
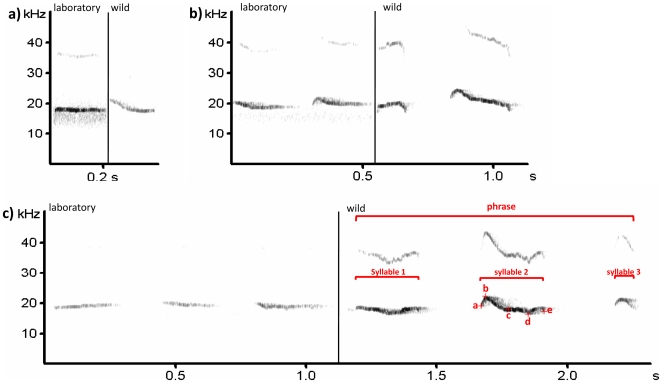
Spectrographs of representative USVs from *P. californicus* in the wild and laboratory. 1 syllable vocalization (1SV; n_laboratory_ = 25, n_wild_ = 6; Audio S1), b) 2SV (n_laboratory_ = 23, n_wild_ = 8; Audio S2), and c) 3SV (n_laboratory_ = 27, n_wild_ = 10; Audio S3), recorded from *P. californicus* in the laboratory and the wild. Frequency resolution for the spectrogram included: FFT length of 512, and a 100% Frame size with a Hamming window. Window overlap was 50%. Prior to spectrograph generation, waveforms were copied into the same file. Annotation as follows with each annotation having an associated frequency (y axis) and time (x axis) value: **a** = start of syllable (*start freq* variable); **b** = maximum frequency of syllable (*max freq* variable); **c** = point of maximum amplitude of syllable (*freq max amp* variable); **d** = minimum frequency of syllable (*min freq* variable); **e** = end of syllable (*end freq* variable). Calculations from these annotations as follows: *duration* of syllable = |time of **a** – time of **e**|; bandwidth of syllable = frequency of **b** - frequency of **d**; *overall modulation* = |frequency of **a**-frequency of **e**|/ duration; *internal modulation* = frequency of **b** - frequency of **d**/ |time between **b** and **d**|.

As with other muroid rodents, all *Peromyscus* species examined to date, including *P. californicus*, produce USVs [18] with 1-, 2-, and 3-syllable vocalizations (henceforth 1SVs, 2SVs, and 3SVs; Figure 1) being the most common USV motifs produced. There are two recent studies that have examined offspring USVs production in laboratory *P. californicus* in relation to offspring development and parental care [9], [11] but none have examined spectral characters of USVs nor production of USVs by adults. Here, for the first time, we report on a comparison of mouse USVs recorded from adults of the same species in the laboratory and the wild. We remotely eavesdropped on vocalizing adult males and females in a laboratory colony and a wild population of the same species. We intentionally did not provide any stimulus for vocalization. We compared 1SVs, 2SVs, and 3SVs produced by *P. californicus* in a laboratory colony and in the wild, to examine spectral and temporal differences between USVs. In addition to being the first comparison of adult mouse USVs from the same species in the laboratory and the wild, this is the first report of spectral and temporal characters of USVs of *P. californicus* in the laboratory, and the first report of USVs recorded from known, individual free living mice in the wild.

## Materials and Methods

### Ethics Statement

Animals in the *Peromyscus* Genetic Stock Center are housed and bred under an approved institutional animal care protocol of the University of South Carolina. The *Peromyscus* Genetic Stock Center is a facility accredited by the Association for the Assessment and Accreditation of Laboratory Animal Care, International, and in accordance with the *Guide for the Care and Use of Laboratory Animals* [19]. For our recordings of California mice at the *Peromyscus* Genetic Stock Center, we did not handle animals nor cause any disturbance beyond what they would normally experience under the approved animal care protocol (Animal Use Protocol # 1321) that covers their welfare. Animal handling techniques in the wild were approved in the University of North Carolina at Greensboro Animal Care Protocol # 07-05. Because our recordings in the wild were taken remotely, and remote sensing equipment was placed off of the focal area containing our resident mice, we did not introduce any human associated odor into the focal area during our recording sessions (average 15.33±3.56 days). Because our remote recording equipment was sensitive to both audible and ultrasonic sound, we know equipment generated noise was minimal and not sufficient to trigger our system.

### Recordings in the Laboratory

USVs were recorded from captive *Peromyscus californicus* at the *Peromyscus* Genetic Stock Center at the University of South Carolina in Columbia, South Carolina, USA. We were interested in comparing spectral characters between adult USVs of the same species, in undisturbed laboratory and free-living contexts, without providing stimuli for vocalizations. Thus, for our laboratory USVs, we recorded vocalizations from a laboratory colony without isolating individuals from the colony. All recordings were made during the night on 2–3 March 2006. The colony of *P. californicus* at the *Peromyscus* Genetic Stock Center was derived from approximately 60 ancestors collected from 1979–1987 in the Santa Monica Mountains, CA. Cages were 16×22×13 cm with aspen shavings for bedding and *ad libitum* water and mouse chow. Room temperature was maintained at 21–22°C and photoperiod was 16L : 8D. A rack of 34 cages of *P. californicus* was isolated from other *Peromyscus* species for recordings. With the exception of 3 cages, all cages contained an adult male-female pair of California mice. Of these 31 pairs, 6 contained a litter of 1–3 pups (mean ± 1 s.d.of litter size = 2.0±0.89 and of litter age 11.83±7.39 ranging from 3–21 days old). *Peromyscus californicus* juveniles are weaned at approximately 60 days of age. The 3 remaining cages contained a single adult female. *Peromyscus californicus* is sexually monomorphic [20] and average (±1 s.d.) mass of breeding condition California mice at the *Peromyscus* Genetic Stock Center is 52.26±8.01 g (n = 20; 10 males and 10 females). Average (±1 s.d.) hindfoot length and tail length of breeding condition California mice at the *Peromyscus* Genetic Stock Center (measured in 2009) is 25.38±0.89 mm (n = 16; 7 males and 9 females) and 120.33±6.65 mm (n = 15; 7 males and 8 females), respectively.

To record vocalizations, a single Pettersson D240x ultrasound detector (Pettersson Elektronik AB, Uppsala, Sweden) capable of recording broadband (10–120 kHz) ultrasound was placed in front of the rack at roughly the middle of all cages 1 m away from the center of the rack. The detector sampled at 307 kHz with 8 bit resolution. The detector was set to continuously record a 3.4 s loop of sound coming through the microphone. Upon detecting any sound in the range of 10–120 kHz the detector was triggered to relay the previous 3.4 seconds of recorded sound with time expanded by a factor of ten, via Sonobat Autorecorder Software (DND Designs) onto an onboard laptop computer sound card (Sigma Tel C-Major Audio). This recording system will be referred to as the Pettersson system.

### Recordings in the Wild

USVs were recorded from free-living *P. californicus* at the Hastings Natural History Reserve in the Santa Lucia Mountains, CA. All recordings were made during the night between 9 February and 25 April 2008 on 17 nights (breeding season is approx January to May). Recordings were made from 12 wild, free-living adult resident California mice (8 females and 4 males). Average mass (±1 s.d.) of individuals we recorded was 39.22±6.50 g (n = 12). Average (±1 s.d.) hindfoot length and tail length of breeding condition of California mice from HNHR (measured from specimens collected from 1986–1990) is 26.52±1.17 mm (n = 42; 21 males and 21 females) and 123.63±7.80 mm (n = 18; 6 males and 12 females), respectively. Details of the study site and animals can be found in [21], [22], [23].

At the study site, we recorded vocalizations with an array (covering approximately 10 m^2^) of 12 Emkay FG Series microphones capable of recording broadband sound (10–120 kHz). Microphones were plugged into an ultrasound recording array with 12 balanced analog inputs (UltraSoundGate 1216H, Avisoft Bioacoustics) and attached via a USB 2.0 interface and RECORDER Software (Avisoft Bioacoustics) to a laptop computer. The microphone array was on the ground in areas where *P. californicus* were resident. The microphones sampled at 250 kHz with 16 bit resolution. This recording system will be referred to as the Avisoft system. We could ensure that USVs were being recorded from *P. californicus* because each of the California mice in, and around, our microphone array was individually outfitted with custom built 0.55 g M1450 mouse transmitters from Advanced Telemetry Systems (ATS). Each transmitter had a unique frequency and California mice were remotely detected using 4 small antennae (Sigflex 15 cm omni-directional) attached to a central receiver (4 MHz R4000), antenna switch box, and data logger (DSU D50410; all from ATS). The receiver was programmed to search continuously for all frequencies of the California mice in the microphone array area. When a frequency was detected at any of the antennae, the receiver recorded the signal strength at all 4 antennae. To determine position of the California mouse based on transmitter signal strength, we made a reference validation grid within the receiver space upon which to compare the signal strength data from radio-collared California mice. The incoming relative signal strength was manually compared to the reference database to assign a position of the California mouse at a particular time within the microphone array area to match to recorded USVs. To ensure that a California mouse without a radio-collar did not produce the USV, a thermal imaging camera (Photon 320 14.25 mm; Flir/Core By Indigo) was used to visualize every mammal in the microphone array space. The camera was suspended ∼10 m above the focal area and wired to a ground-based 30GB hard disk digital video recorder (JVC Everio DVR).

### Terminology

The terms ‘phrase’, ‘syllable’, and ‘motif’ are defined in [18] after [5]. In a previous report that described the first vocalizations from wild *Peromyscus* [18] we defined common USV motifs as ‘# part whistles’ or ‘#PWs’ (ie, 2PW, 3PW, and 4PW; see Figure 1a,b, and c in [18]) reflecting the number of syllables and the whistle like sound of these USV motifs. We have now changed the description of these common motifs from ‘# part whistles’ to ‘# syllable vocalizations’ or ‘#SVs’ reflecting that the parts are syllables and it is not known if these vocalizations are whistles. Therefore, a 2PW in [18] is equivalent to a 2SV herein.

### Spectral and temporal measurements of sound

We extracted time, amplitude, and frequency characteristics from spectrographs rendered by Avisoft-SASLab Pro (Avisoft Bioacoustics). Frequency resolution for the spectrograph included: FFT length of 512, and a 100% Frame size with a Hamming window. Window overlap was 50%. Frequency range of the spectrographic analysis was 125 kHz with a frequency resolution of 488 Hz and a temporal resolution of 1.024 ms. To precisely match the spectrographic analysis bandwidth from both systems, Pettersson system files were imported and re-sampled to the uniform sample rate of 250 kHz. All spectrographic measurements were taken from the fundamental frequency only. For each syllable we measured minimum frequency of syllable, maximum frequency of syllable, peak frequency at the start point, end point, and time point of maximum amplitude of the syllable (annotated in Figure 1c). From these spectrographic measurements, phrase duration, syllable duration and syllable bandwidth were calculated (calculations described in Figure 1c). In addition, to quantify modulation of each syllable, we calculated the slope of the syllable from start point to end point (overall modulation), and from the point of maximum frequency to the point of minimum frequency (internal modulation; calculations and annotations described in Figure 1c).

### Comparison of Recording Systems in the Laboratory and the Wild

To ensure that the Avisoft and Pettersson recording systems did not differ in frequency responses of recorded ultrasound, we set up both systems and presented pure tone sounds, in the range of frequencies of *P. californicus* USVs at a distance comparable to distances in which the vocalizations were measured with each system (approximately 1m). Pure tones of 15, 20, 25, 30, 35, and 40 kHz were presented to the recording system microphones using an AT100 Ultrasonic Transmitter and G'Tools Version 1.6.1 Software (Binary Acoustic Technology). Sound was recorded at each of the frequencies for at least 5 sec. There were 3 replicate sound generations at each frequency. Recording parameters were extracted as described above for both systems and, because they were pure tone signals, included measures of frequency (maximum, minimum, peak, and bandwidth) at the start, end, and maximum amplitude points of the pure tone. To compare the two recording systems we used a two factorial ANOVA on Principal Component (PC) scores of acoustic variables describing frequency and bandwidth of the recorded pure tones using a p<0.05 rejection criterion. We found recording systems to be comparable with no difference in the recording responses of the two recording systems for any of the first three PC axes (accounting for 93% of variation in acoustic variables): PC1 (F_1,24_ = 1.32, p = 0.26), PC2 (F_1,24_ = 1.31, p = 0.26), or PC3 (F_1,24_ = 1.44, p = 0.71). There was no interaction between transmitted frequency and the recording responses of the two systems for PC1 (F_5,24_ = 0.50, p = 0.75), PC2 (F_5,24_ = 0.53, p = 0.75), or PC3 (F_5,24_ = 1.67, p = 0.17).

### Statistical Analysis

For our comparison we used a subset of vocalizations we recorded from the wild from our larger study examining the context of ultrasound production by free-living *P. californicus* [24]. Our subset is representative of the larger data set (total of 223 USVs from 13 females and 5 males [24]) and is scattered through the breeding season in terms of when our vocalizations were recorded. The USVs we selected for this study were simply the first 37 1-, 2-, and 3 SVs that were analyzed using our remote sensing method and we stopped at 37 because this was the number of USVs we had to analyze to have enough 1SVs for statistically valid comparisons (in total we used 9 1SVs, 19 2SVs, and 18 3SVs from 12 of the 13 females and 4 of the 5 males from our larger data set). The subset of vocalizations we used from the laboratory was a randomly selected set of approximately 25 of each 1-, 2-, and 3SVs recorded from the *Peromyscus* Genetic Stock Center.

The five variables that measured frequency (start frequency, end frequency, maximum frequency, minimum frequency, and frequency at maximum amplitude; see Figure 1c) were subjected to a Principal Component analysis to yield a single principal component frequency axis (PC1; details in Table S1). The other 4 variables (duration, overall modulation, internal modulation and bandwidth; see Figure 1c) were not subjected to PC analysis. Therefore, 5 variables (duration, overall modulation, internal modulation, bandwidth and PC1) were used to describe syllables. Additionally, the variable phrase duration was use to describe the phrase. We examined each motif (1-, 2-, and 3SVs) separately because they are unique and do not simply differ in number of syllables [18]. Phrase duration was measured for each motif. For all other variables each syllable was analyzed separately. We tested for homoscedasticity of variance between vocalizations recorded in the wild and in captivity, in the five spectral variables, using a Brown-Forsythe test. Group differences (laboratory *vs.* wild) in the 5 spectral variables were analyzed by Mann-Whitney U tests because data were not normally distributed. Accordingly, descriptive statistics are presented as medians and quartiles. Because of multiple Brown-Forsythe and Mann-Whitney U tests, we use a Bonferroni corrected rejection criterion of p<0.01.

For recordings from the wild, calls of the same individual were averaged, considering individuals as statistically independent data points (analyses without averaging individual calls gave similar results, data not shown). For laboratory calls, we lacked the individual call assignment because we were recording vocalizations with a single microphone from a group of California mice in cages. Therefore, we assumed that the laboratory-recorded vocalizations were made by different adult mice in the laboratory (ie, considering each call as an independent data point). To test the robustness of our results with respect to deviations from this assumption, we also analyzed the data under the extremely conservative assumption that 3, 5, or 7 of the laboratory individuals made all calls. We randomly re-sampled calls 1000 times to calculate averages for the 3, 5, or 7 laboratory individuals to be compared to the calls recorded in the wild. For example, in the 3 re-sample individual condition all laboratory data was randomly assigned to 3 re-sample “individuals.” The calls were averaged for each these re-sample individuals and tested against the calls of wild individuals. The process was repeated 1000 times, providing an indication of the likelihood of finding a difference in calls if only 3 laboratory individuals (in this example) were making the calls. We report how many of these re-sampled data sets result in a significant difference between the wild and laboratory groups at p<0.05. A rejection criterion of p<0.05 provides assurance that any differences seen in Mann-Whitney U tests are biologically meaningful given the reduction in power that accompanies small sample sizes (ie, only 3, 5, or 7 individuals producing vocalizations). Our two approaches (Mann-Whitney U tests on original data and 1000 Mann-Whitney U tests on resampled data assuming three, five, or seven individuals calling in the laboratory) represent two extremes of the probable number of individuals that produced vocalizations and the results were consistent in both cases.

Students t-tests with a rejection criterion of p<0.05 were used to compare body mass, hind foot length and tail length from California mice in the laboratory and the wild. All statistical tests were conducted in Statistica 8 (Statsoft Inc.). Random resamples were conducted in R [25]. All means in text and tables are presented with ±1 s.d. All medians in text and tables are presented with 25% and 75% quartiles.

## Results

We recorded 1SVs, 2SVs and 3SVs for *P. californicus* in the laboratory and wild (Figure 1a, Figure 1b, Figure 1c; Audio S1, Audio S2, Audio S3). The majority of variation in the five variables that measured frequency (start frequency, end frequency, maximum frequency, minimum frequency, and frequency at maximum amplitude; see Figure 1c) was explained by PC1 (Table S1). Descriptive statistics and wild vs laboratory Mann-Whitney U statistics for each motif can be seen in Table 1, Table 2, and Table 3. With the exception of the second syllable of 2SVs, there was no difference in bandwidth of syllables between wild and laboratory USVs with bandwidth ranging from a median of 2.40 to 4.90 kHz regardless of syllable number, motif type, or whether mice were from the wild or the laboratory (Table 1, Table 2, Table 3). Syllable duration differed between wild and laboratory California mice for 1SVs and 3SVs with duration of syllables in the laboratory being consistently longer than in the wild with median syllable durations ranging from 82.0–171.5 ms in the wild vs 176.0–258.0 ms in the laboratory (Table 1, Table 2, Table 3). Syllables recorded from wild California mice were consistently more modulated, both overall and internally, compared with syllables recorded from the wild and this higher modulation was significant in 1SVs (overall modulation) and the first syllable of 3SVs (internal modulation) (Table 1, Table 2, Table 3). In addition, for both 2SVs and 3SVs total duration was longer in California mice recorded in the laboratory (median 452 ms and 669 ms, respectively) than the wild (median 311 ms and 490 ms, respectively).

**Table 1:** Comparison between laboratory- and wild-recorded 1SVs with Mann-Whitney U statistics.

		wild			laboratory			
		(n = 6^c^)			(n = 25)			
Acoustic Variable	Median	Q25	Q75	Median	Q25	Q75	U(b)	P
**Duration (ms)**	140.23	108.00	173.00	197.00	175.60	223.70	18.0	0.0027*
**Start Freq (kHz)**	20.55	20.55	31.70	18.00	17.00	19.50		
**End Freq (kHz)**	18.76	18.50	27.80	16.60	16.10	18.50		
**Max Freq (kHz)**	21.21	20.50	32.20	18.50	18.00	20.00		
**Min Freq (kHz)**	18.33	17.00	26.80	14.60	15.60	17.50		
**Freq Max Amp (kHz)**	19.69	19.50	32.2	17.50	15.60	10.00		
**Bandwidth (kHz)**	3.20	2.50	3.78	2.90	2.00	3.50	53.5	0.2906
**Internal Modulation**	42661.63	20818.38	56074.77	21428.57	16287.88	35409.04	52.0	0.2683
**Overall Modulation**	15739.69	9175.75	23251.49	6993.29	2682.91	10233.44	22.0	0.0061*
**PC1** (a)	−0.28	−2.93	−0.16	0.33	0.01	0.57	19.0	0.0033*

(a) PC1 = First principal component of Frequency Variables.

(b) Mann-Whitney U test statistics for test between captive and wild recorded vocalizations from *P. californicus* on 5 spectral variables. Mann-Whitney U tests significant (*) at p<0.01. Median values (with 25% and 75% quartiles) and samples size from Mann-Whitney U tests are shown.

(c) Data are from 6 individuals and 9 vocalizations.

**Table 2:** Comparison between laboratory- and wild-recorded 2SVs.

		wild			laboratory			
		(n = 8^c^)			(n = 23)			
Acoustic Variable	Median	Q25	Q75	Median	Q25	Q75	U(b)	P
**Syllable 1**								
**Duration (ms)**	157.58	116.00	206.00	258.00	193.00	290.00	42.0	0.0240
**Start Freq (kHz)**	22.36	18.23	32.65	18.50	17.00	19.50		
**End Freq (kHz)**	22.55	19.29	31.00	16.10	14.10	18.00		
**Max Freq (kHz)**	25.64	20.60	33.65	18.50	18.00	20.00		
**Min Freq (kHz)**	21.44	17.88	31.00	15.60	14.10	17.00		
**Freq Max Amp (kHz)**	24.06	19.35	32.40	16.60	15.10	18.50		
**Bandwidth (kHz)**	2.78	2.45	3.18	2.40	1.50	3.90	17.0	0.7675
**Internal Modulation**	47348.27	21236.17	84237.6	24271.84	15537.85	42647.1	54.0	0. 0863
**Overall Modulation**	9935.08	7663.42	12917.15	7584.95	2429.26	12600.81	65.0	0. 2229
**PC1** (a)	−0.67	−2.47	0.16	0.57	0.20	0.86	17.0	0.0007*
**Syllable 2**								
**Duration (ms)**	130.83	112.50	161.39	176.00	117.00	220.00	63.0	0.1905
**Start Freq (kHz)**	25.10	21.58	31.45	19.00	18.00	19.50		
**End Freq (kHz)**	24.18	20.00	29.95	18.50	18.00	19.50		
**Max Freq (kHz)**	28.05	23.40	33.40	19.50	19.00	20.50		
**Min Freq (kHz)**	23.82	18.86	29.70	17.50	17.00	18.50		
**Freq Max Amp (kHz)**	26.56	21.24	33.15	19.00	17.50	20.00		
**Bandwidth (kHz)**	3.81	2.69	4.90	2.40	1.50	2.50	31.0	0.0059*
**Internal Modulation**	60814.94	45261.21	111174.8	30208.33	18248.18	71428.6	44.0	0. 0302
**Overall Modulation**	11839.43	3936.88	23091.41	3685.14	0.00	7421.88	47.0	0. 0422
**PC1**	−1.12	−2.33	−0.24	0.22	0.03	0.38	15.0	0.0005*

(a) PC1 = First principal component of Frequency Variables.

(b) Mann-Whitney U test statistics for test between captive and wild recorded vocalizations from *P. californicus* on 5 spectral variables. Mann-Whitney U tests significant (*) at p<0.01. Median values (with 25% and 75% quartiles) and samples size from Mann-Whitney U tests are shown.

(c) Data are from 8 individuals and 19 vocalizations.

**Table 3:** Comparison between laboratory- and wild-recorded 3SVs.

		wild			laboratory			
		(n = 10^c^)			(n = 27)			
Acoustic Variable	Median	Q25	Q75	Median	Q25	Q75	U(b)	P
**Syllable 1**								
**Duration (ms)**	91.17	67.27	147.00	222.00	171.00	258.00	37.0	0.0008*
**Start Freq (kHz)**	19.00	18.50	25.03	17.50	16.60	19.50		
**End Freq (kHz)**	18.52	18.25	20.13	16.10	15.10	19.00		
**Max Freq (kHz)**	20.95	19.00	25.30	19.00	18.00	20.00		
**Min Freq (kHz)**	17.43	16.10	19.17	15.60	14.60	17.50		
**Freq Max Amp (kHz)**	19.50	17.50	24.37	17.50	16.10	18.50		
**Bandwidth (kHz)**	2.83	2.15	4.40	2.90	2.00	4.40	130.5	0.8777
**Internal Modulation**	71653.12	61538.46	130968.50	28787.88	15017.06	53636.40	35.0	0. 0006*
**Overall Modulation**	20741.52	6103.52	52787.16	8522.73	2838.84	14971.69	73.0	0.0340
**PC1** (a)	0.04	−0.70	0.37	0.57	0.18	0.69	61.0	0.0114
**Syllable 2**								
**Duration (ms)**	171.50	139.33	258.00	237.00	192.00	270.00	74.0	0.0340
**Start Freq (kHz)**	22.90	20.50	30.20	18.00	16.10	19.50		
**End Freq (kHz)**	23.60	18.03	30.20	18.00	17.00	19.50		
**Max Freq (kHz)**	29.75	23.90	33.17	20.00	19.00	20.90		
**Min Freq (kHz)**	22.45	17.50	28.80	16.60	15.10	18.00		
**Freq Max Amp (kHz)**	27.80	20.47	32.37	18.50	17.00	20.00		
**Bandwidth (kHz)**	4.90	4.55	6.60	3.40	2.90	5.30	78.0	0.0513
**Internal Modulation**	30246.91	30192.70	136616.00	27000.00	16853.93	55725.20	85.0	0.0873
**Overall Modulation**	11070.52	8370.54	20451.57	9390.02	4563.38	14088.12	91.0	0.1324
**PC1**	−1.40	−2.39	−0.12	0.34	−0.03	0.59	25.0	0.0002*
**Syllable 3**								
**Duration (ms)**	82.00	58.67	130.57	189.00	131.00	224.00	33.0	0.0005*
**Start Freq (kHz)**	27.38	20.77	31.20	16.60	14.10	19.00		
**End Freq (kHz)**	26.80	19.47	30.87	18.50	16.60	20.00		
**Max Freq (kHz)**	30.00	22.40	33.30	20.00	18.50	20.90		
**Min Freq (kHz)**	26.38	19.30	28.80	15.10	13.10	18.00		
**Freq Max Amp (kHz)**	29.30	21.10	32.53	18.00	17.00	20.00		
**Bandwidth (kHz)**	3.17	2.65	4.10	3.90	2.90	6.30	102.5	0.2664
**Internal Modulation**	92812.14	55539.11	162121.70	79591.84	33333.33	241666.70	123.0	0.6816
**Overall Modulation**	29822.43	18310.55	37364.13	10500.67	4740.60	24190.08	69.0	0.0240
**PC1**	−1.99	−2.38	−0.27	0.49	0.05	0.86	21.0	0.0001*

(a) PC1 = First principal component of Frequency Variables.

(b) Mann-Whitney U test statistics for test between captive and wild recorded vocalizations from *P. californicus* on 5 spectral variables. Mann-Whitney U tests significant (*) at p<0.01. Median values (with 25% and 75% quartiles) and samples size from Mann-Whitney U tests are shown.

(c) Data are from 8 individuals and 19 vocalizations.

**Figure 2:**
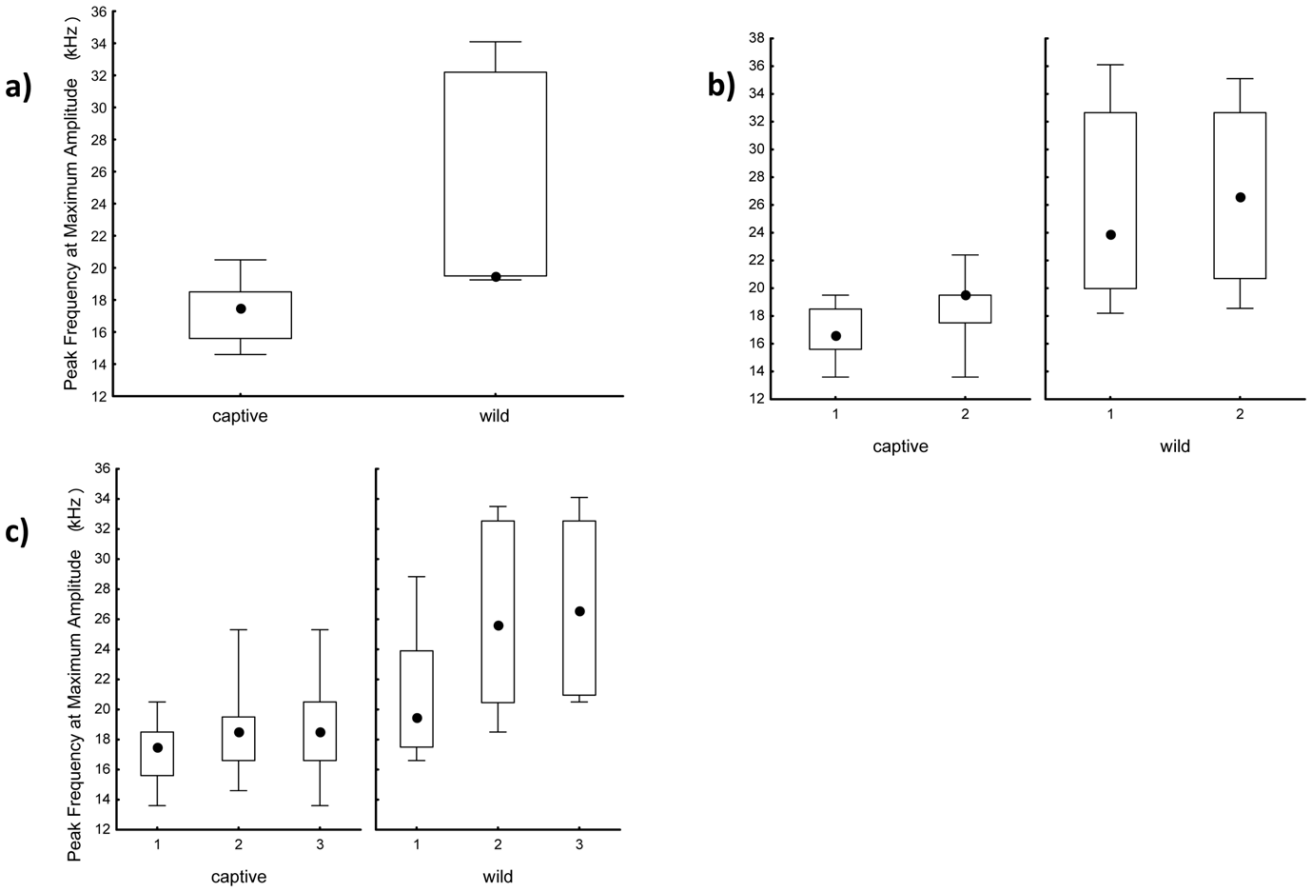
Frequency at maximum amplitude of USVs recorded from *P. californicus* in the wild and laboratory. Box (25% and 75% quartiles) and whisker (ranges) plots of median values (closed circles) of peak frequency of maximum amplitude for a) 1SVs, b) 2SVs, and c) 3SVs recorded from *P. californicus* in captivity and in the wild (sample sizes as in Fig 1).

For every syllable, frequency was higher in USVs recorded from the wild when compared to the laboratory and this difference was significant for every syllable across all motifs except for the first syllable of 3SVs that neared significance (p = 0.0114; Table 1, Table 2, Table 3; Figure 2). Median frequency at maximum amplitude (a representative frequency variable) of 1SVs was 19.69 kHz from wild and 17.50 kHz from laboratory California mice, respectively (Table 1). Median frequency at maximum amplitude of syllable 1 of 2SVs was 24.06 kHz from wild and 16.60 kHz from laboratory California mice, and the second syllable was 26.56 kHz and 19.00 kHz, respectively (Table 2). Median frequency at maximum amplitude of syllable 1 of 3SVs was 19.5 kHz from wild and 17.5 kHz from laboratory California mice, and the second and third syllable was 27.8 kHz and 18.5 kHz, and 29.3 kHz and 18.0 kHz, respectively (Table 3). In addition, the second and third syllables of 3SVs from wild California mice increased in frequency (Figure 1c, Figure 2c) and this interaction between source (wild vs laboratory) and syllable was significant (F_2,105_ = 4.36, p = 0.015; from a two factorial ANOVA run on PC1).

Randomization tests support differences in frequency between wild and laboratory vocalizations and the strength of the difference depended on how many individuals were assumed to be vocalizing in the laboratory colony. Randomization tests that included more than 3 individuals always led to stronger evidence of significance. There were significant differences in frequency for 1SVs in 995 of 1000 resamples, for 2SVs in 999 and 1000 of 1000 resamples, respectively for syllables 1 and 2, and for 3SVs in 999 and 997 of 1000 resamples, respectively for syllables 2 and 3 (3 individuals; Table S2). In addition, randomization tests for other significant duration variables supported differences in duration between wild and laboratory vocalizations. There were significant differences in duration for 1SVs in 979 of 1000 resamples, and for 3SVs in 996 and 993 of 1000 resamples, respectively for syllables 2 and 3 (3 individuals; Table S2). Moreover, the difference in phrase duration for 3SVs was well supported with 1000 of 1000 resamples being significant assuming 3 individuals vocalizing (Table S2). Randomization tests did not support as strongly differences seen in modulation between wild and laboratory vocalizations as only 198 of 1000 resamples for overall modulation of 1SVs were significant (3 individuals; Table S2) and none of the 1000 resamples for internal modulation of syllable 1 of 3SVs were significant (3 individuals vocalizing; Table S2). The difference in bandwidth between syllable 2 of 2SVs of wild and laboratory mice was not supported as strongly with 394 of 1000 resamples being significant (3 individuals vocalizing; Table S2). However, in both these cases, a somewhat less conservative estimate of 7 individuals making vocalizations, did suggest significant differences were present (747 of 1000 resamples significant for overall modulation of 1SV and 988 of 1000 for bandwidth between syllable 2 of 2SV).

The difference in frequency seen between laboratory and wild California mice is not due to differences in body size (larger body sizes tend to have lower frequency vocalizations [26]). Although laboratory California mice have larger mass than wild California mice (*t* = 4.83, df = 23, p<0.0001), their body size is not larger with respect to tail length (wild: n = 18, mean = 123.64 mm; captive: n = 15, mean = 120.33 mm; *t* = 1.31, df = 31, p = 0.20) nor hind foot length as the hind feet of laboratory California mice are approximately 0.6 mm smaller than those of wild California mice (wild: n = 42, mean = 26.53 mm; captive: n = 27, mean = 25.89 mm; *t* = 2.61, df = 66, p = 0.01).

Variance in frequency (PC1) was higher from wild USVs (Table 4; also see quartiles in Figure 2). In addition, variance in overall modulation was higher from wild 2SVs (Table 4). Discriminant functions using the 5 spectral variables were able to classify USVs as wild or laboratory: 1SVs (Wilk's Λ = 0.47, F_5,25_ = 5.71, p<0.0012); 2SVs (Wilk's Λ = 0.41, F_5,56_ = 16.07, p<0.0001); and 3SVs (Wilk's Λ = 0.54, F_5,105_ = 18.13, p<0.0001). Reflecting the higher variance in PC1 for wild-recorded calls, classification success was lower for wild-recorded USVs than laboratory recorded calls: 1SVs 100% and 67%; 2SVs 100% and 69%; and 3SVs 99% and 50%, classification success for USVs recorded in the laboratory and the wild, respectively.

**Table 4:** Homogeneity of variance(b) between laboratory- and wild-recorded 1SVs, 2SVs, and 3SVs.

Motif	Variable		
**1-syllable vocalizations (1SVs)**		**F_1,29_**	**P**
	Duration	0.70	0.39
	Bandwidth	0.01	0.91
	Internal Modulation	0.03	0.87
	Overall Modulation	4.47	0.04
	PC1(a)	5.99	0.02
**2-syllable vocalizations (2SVs)**		**F_3,58_**	**P**
	Duration	0.54	0.66
	Bandwidth	1.49	0.23
	Internal Modulation	1.83	0.15
	Overall Modulation	9.19	0.0005*
	PC1	23.27	<0.00000*
**3-syllable vocalizations (3SVs)**		**F_5,105_**	**P**
	Duration	0.24	0.94
	Bandwidth	0.26	0.93
	Internal Modulation	0.30	0.91
	Overall Modulation	0.79	0.56
	PC1	7.74	0.000005

(a) PC1 = First principal component of Frequency Variables.

(b) Brown-Forsythe Homogeneity of Variance between captive and wild recorded vocalizations from *P. californicus* on spectral variables. Brown-Forsythe tests significant (*) at the adjusted p<0.01. In all cases where significant variance is higher from vocalizations recorded from the wild.

## Discussion

We remotely eavesdropped on vocalizing adult California mice in a laboratory colony and a wild population of the same species without providing any stimulus for vocalization. In both the laboratory colony and the wild population, mice produced 1SVs, 2SVs, and 3SVs as part of their behavior. The similarity in overall motif forms from wild and laboratory California mice demonstrates that production of USVs by laboratory *Peromyscus* is not an artifact of captivity [27], but part of their natural behavioral repertoire. USVs in the wild and laboratory show similar structure in overall motif form, syllable number and bandwidth but not duration nor frequency. Duration of syllables of 1SVs and the first and third syllables of 3SVs were longer in wild California mice than laboratory California mice. There was evidence for 1SVs and syllable 1 of 3SVs from wild California mice to be more modulated than laboratory mice, however this difference was not supported by the randomization tests. Frequencies of USVs from wild California mice were higher than laboratory California mice in syllables of all three motif types. In addition, variance was higher in frequencies of USVs from wild California mice compared with laboratory California mice. Taken together, our results show that USVs from wild California mice are of higher, more variable frequencies.

Because 1SV, 2SV and 3SV motifs were produced by California mice in the wild and the laboratory, our study validates the use of USVs in laboratory rodents as behavioral and phenotypic indicators. However, because motifs differed in frequency and duration depending on whether the motifs were produced by California mice in the wild or the laboratory, our study highlights that particular spectral characteristics of laboratory USVs may not reflect natural conditions. Our results support studies that have suggested captivity may impact acoustic characters of USVs [5], particularly variability and pitch. *Mus* housed in traditional laboratory cages produce less diverse repertoires of USVs with less variability in acoustic features of USVs when compared to *Mus* housed in enriched cages [4].

Our comparison between wild and laboratory vocalizations violated a statistical assumption because we collected data from individuals in the wild but from a group in the laboratory. We assumed that all individuals in the laboratory group were vocalizing in our Mann-Whitney U tests; however the sample potentially contained psuedoreplicated vocalization data from individuals who vocalized multiple times. To test the robustness of our results with respect to this violation, we also analyzed the data under the extremely conservative assumption that only three, five, or seven of the laboratory individuals made all calls using randomization tests and for all variables that describe duration and frequency, randomization tests strongly support differences between wild and laboratory vocalizations (see Table S2).

Related to the assumption that all individuals in the laboratory group were vocalizing is the assumption that only the adults were vocalizing. There were 6 litters among the 31 pairs of California mice in the laboratory and it is possible that some of the vocalizations came from offspring in the nest. However, it is not likely that we recorded USVs from juvenile California mice in the laboratory because they produce USVs in response to being isolated from parents and/or cooled [9], [11] and no such manipulations occurred in our study. Vocalizations from mice in the wild came from both adult males (n = 4) and females (n = 8) and were a representative subset of vocalization from a larger study to examine the situational and demographic context of USV production, and USV characteristics, by wild California mice [24]. Details and comparisons of USVs between adult male and female California mice in the wild are forthcoming in a separate manuscript (Briggs and Kalcounis-Rueppell, unpublished).

Although we show a difference in variability and pitch between wild and laboratory California mice, the particular mechanism(s) that accounts for the differences we found is not known and requires further study. However, it is likely that the difference in frequency between wild and laboratory California mice may be accounted for through mechanisms that either assume 1) wild California mice used to found the laboratory colony vocalized at a lower frequency than the wild California mice we currently study; or 2) wild California mice used to found the laboratory colony vocalized at the same frequency as the mice we currently study and either, through effects of environment or captivity, have lowered the carrier frequency over time or have retained only the lower frequency components. The former may reflect differences in regional dialect between wild populations of *P. californicus*. If this were the case, one would predict that USVs from wild *P. californicus* in the Santa Monica Mountains, where the laboratory California mice were initially collected, would have lower carrier frequencies than those we recorded from *P. californicus* in the Santa Lucia Mountains. The latter may reflect domestication over generations in captivity due to genetic drift or cultural evolution. Generations in captivity can influence behavioral variability in animals [28] and in rodents, attenuation of behavioral variability in captivity has been shown for aggression [29], exploration [30], activity [31], reproduction [32], and morphology [33]. Generations in captivity may have limited variability in California mouse USVs in a way that has been shown for canaries that have smaller song repertoires and fewer variable forms in captivity [34]. There is evidence for genetic and developmental components in USVs in rodents [35], [36], [37] and spectral components of USVs can change in captivity over several generations [38]. In addition, the release of selective pressures from the wild, such as the presence of predators that may have served to maintain the high frequency components, may underlie USV production at lower carrier frequencies in the laboratory.

Regardless of the mechanism, a difference exists and further studies are needed to understand why we see the differences in both USV variability and frequency between wild and laboratory *P. californicus*. In particular, wild USV studies will help in understanding the ultimate reasons and particular mechanisms that account for the difference in spectral and temporal characters. Understanding and appreciating the differences are critical because of the extensive use of rodent ultrasound production as a phenotypic marker in assays of laboratory rats and mice. If social context influences spectral characteristics of USVs, efforts should be made to understand how social context affects USV production. Both *Mus* and *Rattus* readily produce USVs in the laboratory, and the rate of USV production or spectral characters of USVs are used as dependant measures in studies associated with addiction [39], anxiety [40], pain [41], affective states [42], and social processes [43]. For laboratory studies that use USVs as a phenotype, especially those using spectrographic analysis of USVs, the artificial social context of the laboratory environment should be considered in the interpretation of results because we show that both variability in spectral characters, and spectral characters themselves, differ between California mice in the wild and the laboratory.

## Supporting Information

Table S1 Eigenvalues and factor coordinates for variables of PC1 for a) 1 syllable vocalizations, b) 2 syllable vocalizations, and c) 3 syllable vocalizations.
(0.04 MB DOC)

Table S2 Results from randomization tests of significant Mann-Whitney U variables between laboratory- and wild-recorded 1SVs, 2SVs, and 3SVs.
(0.04 MB DOC)

Audio S1 Playback of the the spectrograph in Fig 1a. This is a composite file consisting of a 1SV from a mouse in the laboratory followed by the 1SV from a free-living mouse in the wild. Playback is at 4% of original sound (11.025kHz).
(0.21 MB WAV)

Audio S2 Playback of the the spectrograph in Fig 1b. This is a composite file consisting of a 2SV from a mouse in the laboratory followed by the 2SV from a free living mouse in the wild.Playback is at 4% of original sound (11.025kHz).
(0.80 MB WAV)

Audio S3 Playback of the the spectrograph in Fig 1c. This is a composite file consisting of a 3SV from a mouse in the laboratory followed by the 3SV from a free living mouse in the wild.Playback is at 4% of original sound (11.025kHz).
(1.16 MB MPG)

## References

[1] Sales GD (1999). Ultrasonic communication in rodents.. J. Wildlife Sound Recording Society.

[2] Scattoni M, Crawley J, Ricceri L (2009). Ultrasonic vocalizations: a tool for behavioural phenotyping of mouse models of neurodevelopmental disorders.. Neurosci Biobehav Rev.

[3] Brudzynski SM (2005). Principles of rat communication: quantitative parameters of ultrasonic calls in rats.. Behav Genet.

[4] Portfors C (2007). Types and functions of ultrasonic vocalizations in laboratory rats and mice.. JAALAS.

[5] Holy TE, Guo Z (2005). Ultrasonic songs of male mice.. PLoS.

[6] Smith WJ (1979). Study of Ultrasonic Communication.. Amer Zool.

[7] Kirkland GLJ, Layne JN, Kirkland GLJ, Layne JN (1989). Introduction.. Advances in the Study of *Peromyscus*.

[8] Bredy TW, Lee AW, Meaney MJ, Brown RE (2004). Effect of neonatal handling and paternal care on offspring cognitive development in the monogamous California mouse (*Peromyscus californicus*).. Horm Behav.

[9] Wright SL, Brown RE (2004). Sex differences in ultrasonic vocalizations and coordinated movement in the California mouse (*Peromyscus californicus*).. Behav Process.

[10] Vieira ML, Brown RE (2003). Effects of the presence of the father on pup development in California mice (*Peromyscus californicus*).. Dev Psychobiol.

[11] Vieira ML, Brown RE (2002). Ultrasonic vocalizations and ontogenetic development in California mice (*Peromyscus californicus*).. Behav Process.

[12] Lee AW, Brown RE (2007). Comparison of medial preoptic, amygdala, and nucleus accumbens lesions on parental behavior in California mice (*Peromyscus californicus*).. Physiol Behav.

[13] Bester-Meredith JK, Martin PA, Marler CA (2005). Manipulations of vasopressin alter aggression differently across testing conditions in monogamous and non-monogamous *Peromyscus* mice.. Aggressive Behav.

[14] Davis ES, Marler CA (2004). C-fos changes following an aggressive encounter in female California mice: A synthesis of behavior, hormone changes and neural activity.. Neuroscience.

[15] Fuxjager MJ, Mast G, Becker EA, Marler CA (2009). The ‘home advantage’ is necessary for a full winner effect and changes in post-encounter testosterone.. Horm Behav.

[16] Trainor BC, Finy MS, Nelson RJ (2008). Rapid effects of estradiol on male aggression depend on photoperiod in reproductively non-responsive mice.. Horm Behav.

[17] Trainor BC, Finy MS, Nelson RJ (2008). Paternal aggression in a biparental mouse: Parallels with maternal aggression.. Horm Behav.

[18] Kalcounis-Rueppell MC, Metheny JD, Vonhof MJ (2006). Production of ultrasonic vocalizations by *Peromyscus* mice in the wild.. Front Zool.

[19] National Research Council (1996). Guide for the care and use of laboratory animals.

[20] Merritt J (1978). *Peromyscus californicus*.. Mammalian Species.

[21] Kalcounis-Rueppell MC, Millar JS (2002). Partitioning of space, food, and time by syntopic *Peromyscus boylii* and *P. californicus*.. J Mammal.

[22] Ribble DO (1991). The monogamous mating system of *Peromyscus californicus* as revealed by DNA fingerprinting.. Behav Ecol Sociobiol.

[23] Ribble DO (1992). Dispersal in a monogamous rodent, *Peromyscus californicus*.. Ecology.

[24] Briggs J (2009). The individual context of ultrasonic vocalizations in wild monogamous California mice (*Peromyscus californicus*).

[25] R Development Core Team (2008). R: A Language and Environment for Statistical Computing.. http://www.R-project.org.

[26] Bradbury J, Vehrencamp S (1998). Principles of Animal Communication.

[27] Wolff JO (2003). Laboratory studies with rodents: Facts or artifacts?. Bioscience.

[28] Price EO (1999). Behavioral development in animals undergoing domestication.. Appl Anim Behav Sci.

[29] Kunzl C, Kaiser S, Meier E, Sachser N (2003). Is a wild mammal kept and reared in captivity still a wild animal?. Horm Behav.

[30] Augustsson H, Dahlborn K, Meyerson BJ (2005). Exploration and risk assessment in female wild house mice (*Mus musculus musculus*) and two laboratory strains.. Physiol Behav.

[31] Gattermann R, Johnston RE, Yigit N, Fritzsche P, Larimer S (2008). Golden hamsters are nocturnal in captivity but diurnal in nature.. Biol Lett.

[32] Botten J, Ricci R, Hjelle B (2001). Establishment of a deer mouse (*Peromyscus maniculatus rufinus*) breeding colony from wild-caught founders: comparison of reproductive performance of wild-caught and laboratory-reared pairs.. Comp Med.

[33] McPhee ME (2004). Morphological change in wild and captive oldfield mice *Peromyscus polionotus subgriseus*.. J Mammal.

[34] Guttinger HR (1985). Consequences of domestication on the song structures in the canary.. Behaviour.

[35] Thornton LM, Hahn ME, Schanz N (2005). Genetic and developmental influences on infant mouse ultrasonic calling. III. Patterns of inheritance in the calls of mice 3–9 days of age.. Behav Genet.

[36] Panskepp J, Jochman K, Kim J, Koy J, Wilson E (2007). Affiliative behavior, ultrasonic communication and social reward are influenced by genetic variation in adolescent mice.. PLoS ONE.

[37] Wohr M, Dahlhoff M, Wolf E, Holsboer F, Schwarting RKW (2008). Effects of Genetic Background, Gender, and Early Environmental Factors on Isolation-Induced Ultrasonic Calling in Mouse Pups: An Embryo-Transfer Study.. Behav Genet.

[38] Brunelli SA, Vinocur DD, Soo-Hoo D, Hofer MA (1997). Five generations of selective breeding for ultrasonic vocalization (USV) responses in N:NIH strain rats.. Dev Psychobiol.

[39] Vivian JA, Miczek KA (1999). Interactions between social stress and morphine in the periaqueductal gray: effects on affective vocal and reflexive pain responses in rats.. Psychopharmacology (Berl).

[40] Blanchard DC, Griebel G, Blanchard RJ (2001). Mouse defensive behaviors: pharmacological and behavioral assays for anxiety and panic.. Neurosci Biobehav Rev.

[41] Calvino B, Besson JM, Boehrer A, Depaulis A (1996). Ultrasonic vocalization (22–28 kHz) in a model of chronic pain, the arthritic rat: effects of analgesic drugs.. Neuroreport.

[42] Knutson B, Burgdorf J, Panksepp J (2002). Ultrasonic Vocalizations as indices of affective states in rats.. Psychol Bull.

[43] Moles A, Costantini F, Garbugino L, Zanettini C, D'Amato FR (2007). Ultrasonic vocalizations emitted during dyadic interactions in female mice: A possible index of sociability?. Behav Brain Res.

