# Supplementary material for: Differences in Ultrasonic Vocalizations between Wild and Laboratory California Mice (Peromyscus californicus)
Source: PLoS One. 2010 Apr 1;5(4):e9705. doi: 10.1371/journal.pone.0009705 (PMC2848568; doi:10.1371/journal.pone.0009705)
Supplement: Table S1 — Eigenvalues and factor coordinates for variables of PC1 for a) 1 syllable vocalizations, b) 2 syllable vocalizations, and c) 3 syllable vocalizations. (0.04 MB DOC) [file pone.0009705.s001.doc]

**a)**

| **1 syllable vocalization** | |
| --- | --- |
|  | **PC1** |
| **Acoustic Variable** | **(96.93%)** |
| Start Freq (kHz) | -0.98 |
| End Freq (kHz) | -0.98 |
| Max Freq (kHz) | -0.99 |
| Min Freq (kHz) | -0.99 |
| Freq Max Amp (kHz) | -0.99 |

**b)**

| **2syllable vocalization** | |
| --- | --- |
|  | **PC1** |
| **Acoustic Variable** | **(97.29%)** |
| Start Freq (kHz) | -0.97 |
| End Freq (kHz) | -0.99 |
| Max Freq (kHz) | -0.99 |
| Min Freq (kHz) | -0.99 |
| Freq Max Amp (kHz) | -0.99 |

| **3 syllable vocalization** | |
| --- | --- |
|  | **PC1** |
| **Acoustic Variable** | **(93.74%)** |
| Start Freq (kHz) | -0.94 |
| End Freq (kHz) | -0.96 |
| Max Freq (kHz) | -0.97 |
| Min Freq (kHz) | -0.98 |
| Freq Max Amp (kHz) | -0.98 |

**c)**
