# Supplementary material for: Differences in Ultrasonic Vocalizations between Wild and Laboratory California Mice (Peromyscus californicus)
Source: PLoS One. 2010 Apr 1;5(4):e9705. doi: 10.1371/journal.pone.0009705 (PMC2848568; doi:10.1371/journal.pone.0009705)
Supplement: Table S2 — Results from randomization tests of significant Mann-Whitney U variables between laboratory- and wild-recorded 1SVs, 2SVs, and 3SVs. (0.04 MB DOC) [file pone.0009705.s002.doc]

| **Motif** |  | **number of significant tests/1000 randomizations** | | |
| --- | --- | --- | --- | --- |
| Syllable Number | **Significant Variable from Mann Whitney U Test** | **assume 3 individuals** | **assume 5 individuals** | **assume 7 individuals** |
| **1-syllable** **vocalizations** |  |  |  |  |
| Syllable 1 | Duration | 979 | 986 | 998 |
|  | Overall Modulation | 198 | 436 | 747 |
|  | PC1^(a)^ | 995 | 982 | 996 |
| **2-syllable vocalizations** |  |  |  |  |
| Syllable 1 | PC1 | 999 | 1000 | 1000 |
| Syllable 2 | Bandwidth | 394 | 958 | 988 |
|  | PC1 | 1000 | 1000 | 1000 |
| **3-syllable vocalizations** |  |  |  |  |
|  | Phrase Duration | 1000 | 1000 | 1000 |
| Syllable 1 | Duration | 996 | 999 | 1000 |
|  | Internal Modulation | 0 | 0 | 117 |
| Syllable 2 | PC1 | 999 | 1000 | 1000 |
| Syllable 3 | Duration | 993 | 1000 | 1000 |
|  | PC1 | 997 | 1000 | 1000 |

1. PC1 = First principal component of Frequency Variables
